# The Novel LncRNA AK035396 Drives Cardiomyocyte Apoptosis Through Mterf1 in Myocardial Ischemia/Reperfusion Injury

**DOI:** 10.3389/fcell.2021.773381

**Published:** 2021-11-08

**Authors:** Zhaoyan Xu, Yuanxi Mo, Xinyi Li, Wanzi Hong, Sisi Shao, Yaoxin Liu, Fen Shu, Lei Jiang, Ning Tan

**Affiliations:** ^1^ Department of Cardiology, the Second School of Clinical Medicine, The First People Hospital of Foshan, Southern Medical University, Guangzhou, China; ^2^ Department of Cardiology, Guangdong Provincial Key Laboratory of Coronary Heart Disease Prevention, Guangdong Cardiovascular Institute, Guangdong Provincial People’s Hospital, Guangdong Academy of Medical Sciences, Guangzhou, China; ^3^ School of Medicine, South China University of Technology, Guangzhou, China; ^4^ Guangdong Provincial Geriatrics Institute, Guangdong Provincial People’s Hospital, Guangdong Academy of Medical Sciences, Guangzhou, China

**Keywords:** Mterf1, apoptosis, ischemia/reperfusion, lncRNA, mitochondrial function

## Abstract

**Background:** Myocardial ischaemia/reperfusion (I/R) injury is still a major challenge in clinical treatment. The role of long non-coding RNA (lncRNA) in the regulation of myocardial I/R injury still needs to be elucidated.

**Methods:** The primary isolated neonatal mousse cardiomyocytes and adult mice were used to construct a myocardial ischemia-reperfusion model. qRT-PCR is used to verify gene expression in myocardial tissue and myocardial cells. The effect of AK035396 in primary cardiomyocytes and mouse myocardium was confirmed by TUNEL staining and *in vitro* flow cytometry experiments. RNA pulldown and Western blot were used to identify AK035396 interacting proteins. The expression of apoptosis-related proteins was identified by qRT-PCR and Western blot.

**Results:** *In vivo* and *in vitro* MIRI models, AK035396 was up-regulated after myocardial infarction. Functional studies have shown that knockdown of AK035396 reduces the apoptosis of primary cardiomyocytes and mouse myocardial tissue. AK035396 directly interacts with Mterf1 and inhibits the level of Mterf1. Further experiments have shown that inhibiting Mterf1 will promote the expression of mitochondrial genes COXII and CYTb and cause cell apoptosis.

**Conclusion:** AK035396 plays an important role in myocardial ischaemia-reperfusion injury by regulating the Mterf1-COXII/CYTb pathway.

## Introduction

Acute myocardial infarction (AMI), which is caused by coronary stenosis or acute arterial occlusion, has a very high mortality and disability rate. Reperfusion therapy is currently one of the most important methods for curing patients with acute myocardial infarction (Zidan et al., 2016; Reed et al., 2017). However, reperfusion therapy may cause ischaemia-reperfusion injury, in which apoptosis plays a crucial role, although its exact mechanism is still unclear.

Long noncoding RNAs (lncRNAs) are transcribed RNA molecules >200 nucleotides in length that lack a specific open reading frame and have no significant protein-coding potential; however, lncRNAs play important roles in the life cycle, proliferation, migration and metabolism of cells (Wei and Wang, 2017; Zhao et al., 2018; Qian et al., 2019). Therefore, lncRNAs are very likely to play important roles in I/R. After preliminary experiments, we found that the long noncoding RNA AK035396 was significantly increased in the experimental group. In addition, bioinformatics analysis indicated that AK035396 may be associated with Mterf1 (mitochondrial transcription termination Factor 1).

In-depth study of mitochondrial gene transcription mechanisms and mitochondrial diseases has shown that Mterf1, which plays an important regulatory role in mitochondria, is of great importance. Mterf1 is composed of 343 amino acid residues and contains two independent DNA binding regions and three leucine zippers. This factor is present in the form of monomers on MtDNA and plays a functional role (Fernandez-Silva et al., 1997; Terzioglu et al., 2013; Chen et al., 2014). MtDNA has a 28 BP sequence at the junction of the 16S rRNA coding gene and tRNALeu (UUR) coding gene. Mterf1 can bind at this site, which can significantly reduce the affinity of RNA polymerase for the transcription template and terminate H-strand transcription in advance (Martin et al., 2005; Asin-Cayuela and Gustafsson, 2007; Terzioglu et al., 2013; Falkenberg, 2018; Hillen et al., 2018).

Apoptosis is a highly regulated cell death process that is involved in a variety of biological processes, such as cancer, development, and ischaemic diseases (Domingos and Steller, 2007; Wong, 2011; Li and Liu, 2018). There are many intrinsic signalling pathways that initiate apoptosis, and apoptosis initiated by the mitochondrial pathway is important (Li and Liu, 2018). COXII and CYTb are two important proteins associated with mitochondrial apoptosis (Shi et al., 2015; Wang et al., 2016). These factors are transcribed in mitochondria and released into the cytoplasm to induce apoptosis.

This study shows that AK035396 plays a key role in regulating I/R-induced cardiomyocyte apoptosis and elucidates its regulatory role in the AK035396/Mterf1/COXII/CYTb axis. Therefore, these results provide new insights into cardiomyocyte apoptosis during I/R.

## Materials and Methods

### Experimental Animals and I/R Models

Male C57BL/6 mice (6–8 weeks old) were purchased from Beijing Weitong Lihua. All mice were randomly divided into four groups (6 mice per group): NC (sham operation) group, I/R (ischaemia reperfusion) group, I/R + Scramble, and I/R + AK035396 shRNA. The experimental protocols were approved by the Medical Research Ethics Committee of Guangdong Provincial People’s Hospital and were implemented in accordance with the National Institutes of Health’s “Laboratory Animal Care and Use Guidelines”. The mice had unlimited access to water and food before the experiment. Before modelling, we anaesthetized the mice by injecting 0.15% Pentobarbital and fixed the mice *via* tracheal intubation with a small animal respirator. Next, we made a longitudinal incision from the third to fourth ribs to expose the heart. Then, a 5–0 Proline suture (2 cm) was placed around the root of the left anterior descending coronary artery (LAD). After 30 min of occlusion, the sutures were released, and the LAD was perfused for an additional 120 min.

### Isolation of Primary Cardiomyocytes

Primary cardiomyocytes (CMs) from the mouse neonatal heart were isolated. In short, 1–3-day-old new-born mice were sacrificed by decapitation. The ventricle was immediately excised and digested in ice-cold PBS (Ca 2+ and Mg 2 + free) with 0.125% trypsin and 0.05% type I collagenase (Thermo Fisher Scientific Inc.) solutions. To enrich CMs, the cells were preseeded for 1.5 h to remove nonmuscle cells. CMs were cultured on collagen-coated tissue culture dishes in cardiomyocyte culture medium (CMM, ScienCell Research Laboratories Inc.) containing 10% FBS. All cells were kept in a 37°C, 5% CO_2_ incubator. CMs in the I/R group was exposed to hypoxia for 30 min, followed by reoxygenation for 4 h to establish an ischemia-reperfusion model.

### Quantitative Real-Time PCR

TRIzol™ reagent (Invitrogen, Carlsbad, CA, United States) was used to isolate total RNA. Two micrograms of total RNA was used for reverse transcription using the PrimeScript® first Strand Synthesis Kit (TaKaRa, Tokyo, Japan). Real-time RT–qPCR was performed using a QuantiTect SYBR® Green RT–PCR kit (QIAGEN, Düsseldorf, Germany). The primer sequences for RT–qPCR are shown in Table 1. GAPDH was used for standardization. The relative expression of the lncRNA AK035396, Mterf1, Caspase3, Caspase9, COX II, and CYTb was determined by the 2^−ΔΔCt^ method.

**TABLE 1:** Primers for qRT-PCR.

AK035396	Forward Primer	TGT​ATT​GGT​CTT​GCT​TGT​GTT
	Reverse Primer	ACA​GTC​GTC​AGT​CAT​CAG​T
Mterf1	Forward Primer	GCA​CAT​CCA​GCA​TTG​CTC​TG
	Reverse Primer	AAA​CTG​GAA​CCA​GTG​CCA​CA
Caspase3	Forward Primer	ATT​CAA​AGG​ACG​GGT​CGT​GG
	Reverse Primer	GTG​GAA​AGT​GGA​GTC​CAG​GG
Caspase9	Forward Primer	ACA​GAT​GGA​TGC​TCC​GTG​TC
	Reverse Primer	CAA​GGT​CCT​GCC​TTG​AGA​GG
COXII	Forward Primer	ACC​TGG​TGA​ACT​ACG​ACT​GC
	Reverse Primer	CTA​GGG​AGG​GGA​CTG​CTC​AT
CYTb	Forward Primer	GGC​TAC​GTC​CTT​CCA​TGA​GG
	Reverse Primer	AGC​GAA​GAA​TCG​GGT​CAA​GG

### Construction of shRNA-AK035396 Lentiviral Vectors

Specific short hairpin RNAs and scrambled oligonucleotides of murine AK035396 were synthesized by GenePharma (Shanghai, China). Then, 20 μg of AK035396 shRNA or scrambled shRNA was inserted into the BLOCK-iT™ Lentiviral RNAi Expression System (Invitrogen). Briefly, 293T cells (Thermo Fisher, Shanghai, China) were cotransfected with lentiviral vectors and packaging vectors. The supernatant was collected after 48 and 72 h. The viral supernatant was concentrated with lentivirus concentration reagent (Biomiga, CA, United States). High-titre virus (1 × 10^9^ PFU/ml) was resuspended in PBS. The animals were anaesthetized with 2% isoflurane, and an incision was made between the left fourth and fifth ribs to expose the heart. A 30-gauge needle was used to inject 10 μl of concentrated lentivirus and AK035396 shRNA into the apex and anterior wall of the heart. The other group of mice was injected with 10 μl of lentiviral hybrid shRNA and used as the Scramble group.

### TUNEL Staining

After the adult mice were reperfused, the heart was quickly excised and cut into 5 μm-thick sections, while neonatal mouse cardiomyocytes were used to prepare cell slides, which were permeabilized with 2% Triton-x-100. Terminal deoxynucleotidyl transferase dUTP nick end labelling (TUNEL) was used to evaluate cardiomyocyte apoptosis in heart slices and cell slides. The TUNEL mixture contained 50 μl of enzyme solution and 450 μl of labelling solution. Heart slices were incubated with 50 μl of the TUNEL mixture at 37°C for 1 h. The sections were washed three times in phosphate-buffered saline (PBS) and stained with DAPI. After being washed 3 times with PBS, the sections were observed under a fluorescence microscope. The apoptotic ratio is the number of apoptotic cells (green)/total number of cells (blue) × 100%.

### Western Blot Analysis

RIPA lysis buffer (Beyotime, Shanghai, China) was used to obtain total proteins, 100 μg of which was separated by SDS-polyacrylamide gel electrophoresis and transferred to a polyvinylidene fluoride (PVDF) membrane. TBST containing 5% skimmed milk was added to the membrane and incubated for 1 h. Then, the membrane was incubated with primary antibodies, including anti-Mterf1 (Sigma–Aldrich), anti-COXII (Abcam), anti-CYTb (Abcam), and anti-GAPDH (Abcam), overnight at 4°C. After the membrane was washed 3 times in TBST, the membrane was incubated with anti-rabbit IgG H and L (HRP) secondary antibodies (ab6721, 1:2000, Abcam) at room temperature for 1.5 h.

### Pull Down Assay

A total of 1 × 10^7^ mouse primary cardiomyocytes were harvested, lysed and sonicated. The AK035396 probe was added and incubated with C-1 magnetic beads (Life Technologies) at 25°C for 2 h to generate probe-coated beads. Cell lysates were incubated with the AK035396 probe or oligo probe at 4°C overnight. After being washed with wash buffer, the RNA mix bound to the beads was eluted and extracted with an RNeasy Mini Kit (QIAGEN) for RT–PCR or real-time PCR analysis.

### Apoptosis Assay

Apoptosis was determined by the translocation of phosphatidylserine to the cell surface using an Annexin V Alexa Fluor647/PI/apoptosis detection kit (Si Zhengbai. Co. Ltd., China). Primary neonatal mouse cardiomyocytes with stable knockdown of lncRNA AK035396 and their negative control cells were harvested, washed twice with cold PBS, and then resuspended in Annexin V-647/PI for 10 min in the dark. Cell apoptosis was analysed by using Cell Quest software on a FACSAria flow cytometer (BD Company, United States). Fluorescence was detected at an excitation wavelength of 647 nm.

### Statistical Analysis

The data were analysed by SPSS 17.0 statistical analysis software (SPSS Inc., Chicago, Illinois, United States) and are expressed as the mean ± standard deviation (m ± SD). Statistically significant differences between groups were determined by ANOVA. Two-tailed Student’s t-tests were used to evaluate the differences between the groups and their respective controls. *p* < 0.05 was considered to be significant.

## Results

### AK035396 is Upregulated in Myocardial Ischaemia-Reperfusion

**FIGURE 1:**
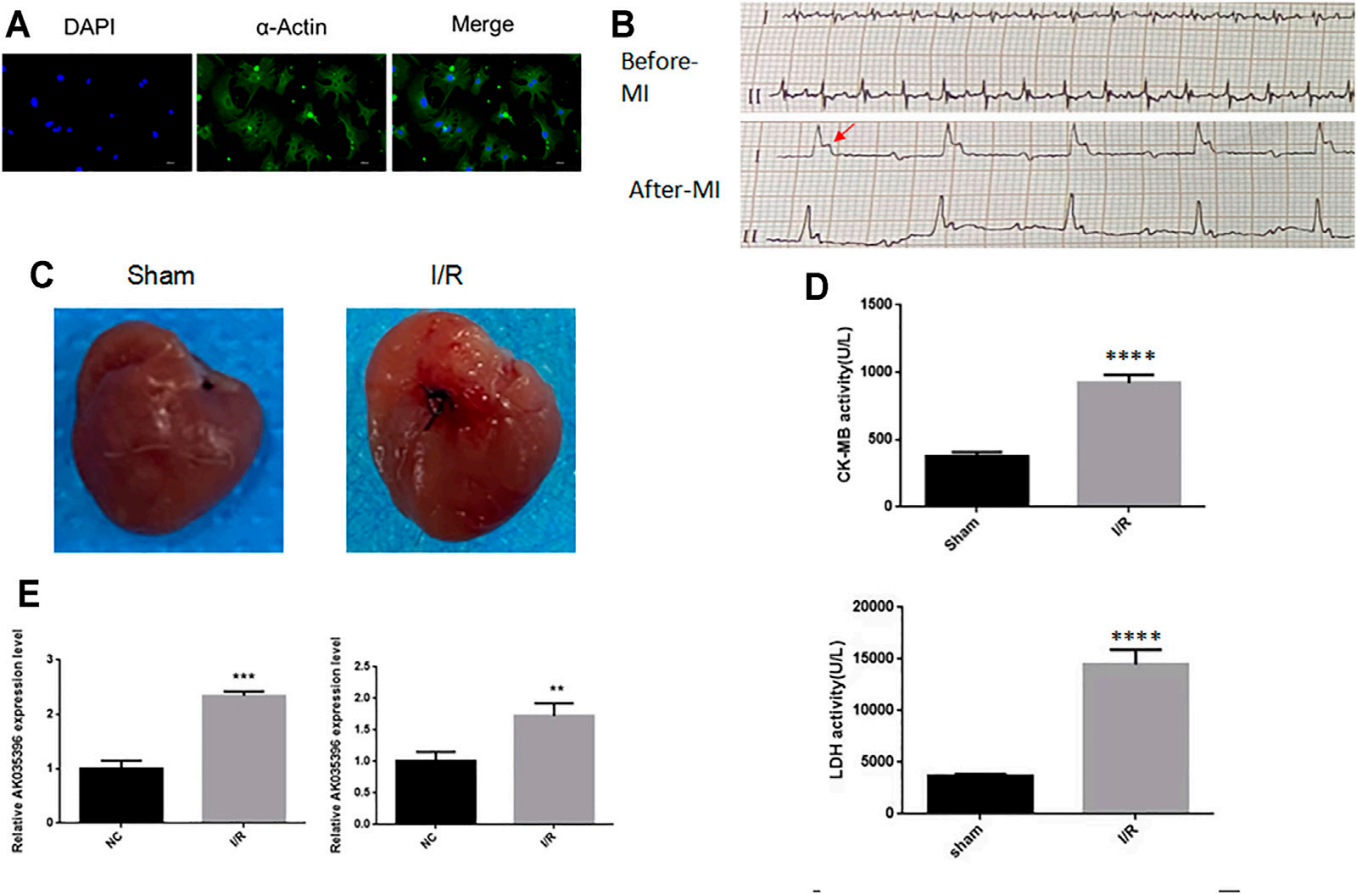
The expression of AK035396 in the ischaemia-reperfusion model **(A)** Primary murine cardiomyocytes were isolated, and the purity of the cells was evaluated with α-Actin (Blue, DAPI) (Green, α-Actin). **(B)** Electrocardiogram of the mouse myocardial infarction model. The arrow indicates abnormal T waves. **(C)** Myocardial changes before and after myocardial infarction modelling in mice. **(D)** ELISA results showing that CK-MB and LDH were increased in mice with myocardial infarction. **(E)** After the hypoxia-reperfusion model was established in primary cardiomyocytes **(left)** and mice **(right)**, the expression of AK035396 was measured by qRT–PCR.

First, we established a myocardial ischaemia-reperfusion model in neonatal mouse primary cardiomyocytes to determine the effect of AK035396 on myocardial ischaemia-reperfusion. To verify the expression of AK035396 in the ischaemia-reperfusion model, we isolated neonatal mouse cardiomyocytes *in vitro* (Figure 1A) and exposed the cells to hypoxia at 37°C for 30 min, followed by reoxygenation for 4 h. Compared with that in the control group, the apoptosis rate in the I/R group was significantly increased. Furthermore, we established a mouse myocardial infarction reperfusion model. ECG and analysis of the myocardial enzymes CK-MB and LDH showed that the mouse myocardial infarction model was successfully established (Figures 1B–D). After 30 min of ligation of the left anterior descending branch, perfusion was restored for 2 h to establish the I/R model. These results indicate that we successfully established an ischaemia-reperfusion model. Then, we verified the expression of AK035396 in cardiomyocytes by qRT–PCR, and the results showed that AK035396 was significantly upregulated in the I/R group (Figure 1E). Furthermore, we examined the changes in AK035396 in mice. By constructing a mouse myocardial ischaemia-reperfusion model, we found that the expression of AK035396 in the I/R group increased (Figure 1E), which was consistent with the *in vitro* results.

### Identification and Characterization of AK035396

**FIGURE 2:**
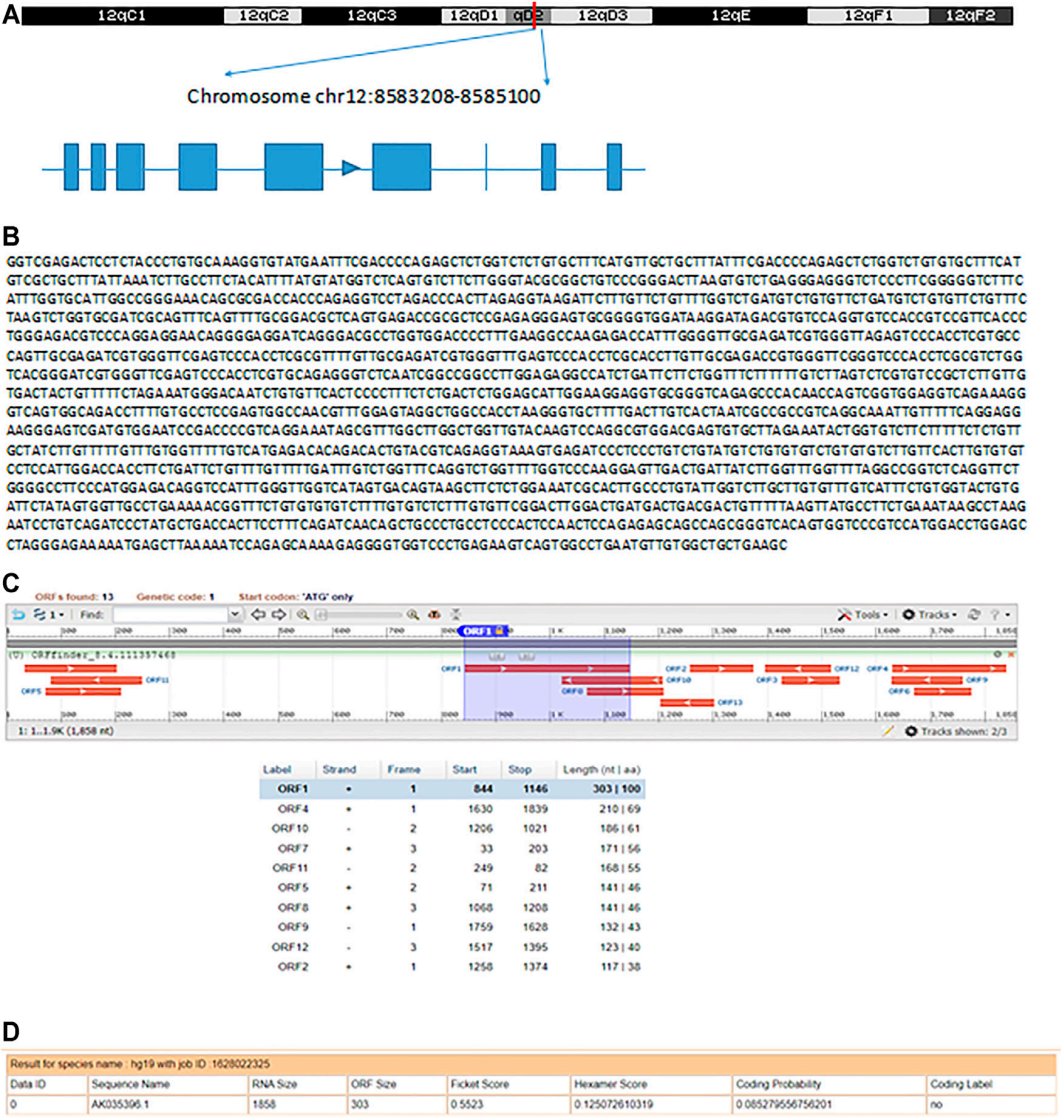
Identification and characterization of AK035396. **(A)** Schematic diagram of AK035396 from Ensembl RefSeq and RACE. Blue boxes: exons annotated by NCBI RefSeq. Blue lines: introns. The arrows on the blue lines indicate transcriptional directions. **(B)** The full-length sequence of the AK035396 transcript. **(C, D)** ORF finder software **(C)** and CPAT software. **(D)** Prediction of the protein-coding potential of AK035396.

Based on the UCSC Genome Browser, we found that AK035396 is located on murine chromosome 12D2 and composed of nine exons, as annotated by Ensembl RefSeq (exon 1, 55 bp; exon 2, 9 bp; exon 3, 50 bp; exon 4, 104 bp; exon 5, 253 bp; exon 6, 292 bp; exon 7, 4 bp; exon 8, 53 bp; exon 9, 21 bp) (Figure 2A). Based on the Sanger sequencing results, the full-length AK035396 transcript was found to be 1,841 nt (Figure 2B). However, the ORF finder from NCBI2 failed to predict a protein based on the AK035396 sequence, as determined by RACE (Figure 2C). The CPAT database also showed that AK035396 had limited protein-coding potential (coding probability = 0.085279556) (Figure 2D).

### AK035396 Promotes Myocardial Cell Apoptosis During Myocardial I/R

**FIGURE 3:**
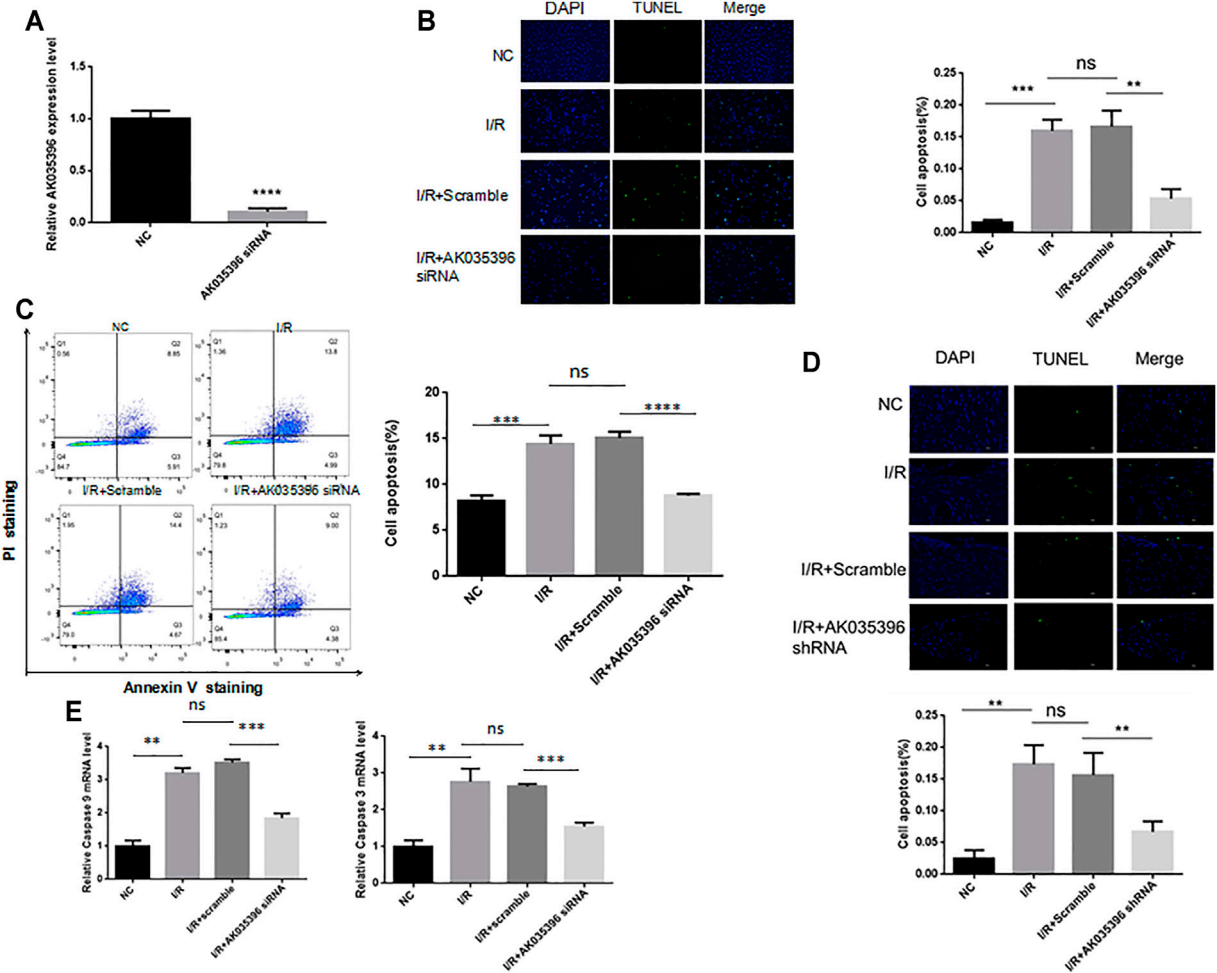
Effect of AK035396 on apoptosis after myocardial ischaemia reperfusion. Primary cardiomyocytes were divided into four groups: NC, I/R, I/R + Scramble and I/R + AK035396 siRNA. C57BL/6 mice were divided into four groups: NC, I/R, I/R + Scramble and I/R + AK035396 shRNA. A TUNEL apoptosis detection kit was used to measure apoptosis in each group. **(A)** The knockdown efficiency of AK035396 siRNA was determined by qRT–PCR. **(B)** Primary cardiomyocyte apoptosis was measured by TUNEL staining (green). The nucleus was counterstained with 4′,6-diamidino-2-phenylindole (DAPI, blue). **(C)** Flow cytometry was used to measure apoptosis in primary cardiomyocytes. **(D)** Apoptosis in mouse myocardial was determined by TUNEL staining (green). The nucleus was counterstained with 4′,6-diamidino-2-phenylindole (DAPI, blue). NC: Normal control; I/R: Ischaemia reperfusion. **(E)** The expression of caspase3 and caspase9 was measured by qRT–PCR.

To explore the role of AK035396 in myocardial ischaemia-reperfusion, we used small interfering RNA (siRNA) to modify primary mouse cardiomyocytes and inhibit the expression of AK035396 (Figure 3A). The qRT–PCR results showed that compared with that the NC group, the expression of AK035396 in siRNA-transfected cells was significantly reduced. TUNEL staining showed that myocardial ischaemia-reperfusion could induce cardiomyocyte apoptosis, and inhibiting AK035396 could reverse cardiomyocyte apoptosis (Figure 3B). In addition, flow cytometry showed that apoptosis in the I/R group was significantly increased, and after knocking down AK035396, apoptosis in the I/R group was reduced (Figure 3C). Caspase3 and caspase9 are classic proteins in the apoptosis signalling pathway. These proteins were significantly upregulated in the I/R group. After interference with AK035396, the expression of both decreased, indicating that inhibiting AK035396 could reduce apoptosis (Figure 3D). Furthermore, we established a mouse ischaemia-reperfusion model and examined apoptosis in myocardial tissue by TUNEL staining. Consistent with the results in primary cardiomyocytes from neonatal mice, myocardial apoptosis in the I/R group was higher than that in the sham operation group, while myocardial apoptosis was significantly reduced in mice with shRNA-mediated AK035396 knockdown (Figure 3E). Overall, these results indicate that the overexpression of AK035396 during ischaemia-reperfusion exacerbates the apoptosis of cardiomyocytes.

### AK035396 Regulates Mitochondrial-Mediated Apoptosis by Regulating Mterf1

**FIGURE 4:**
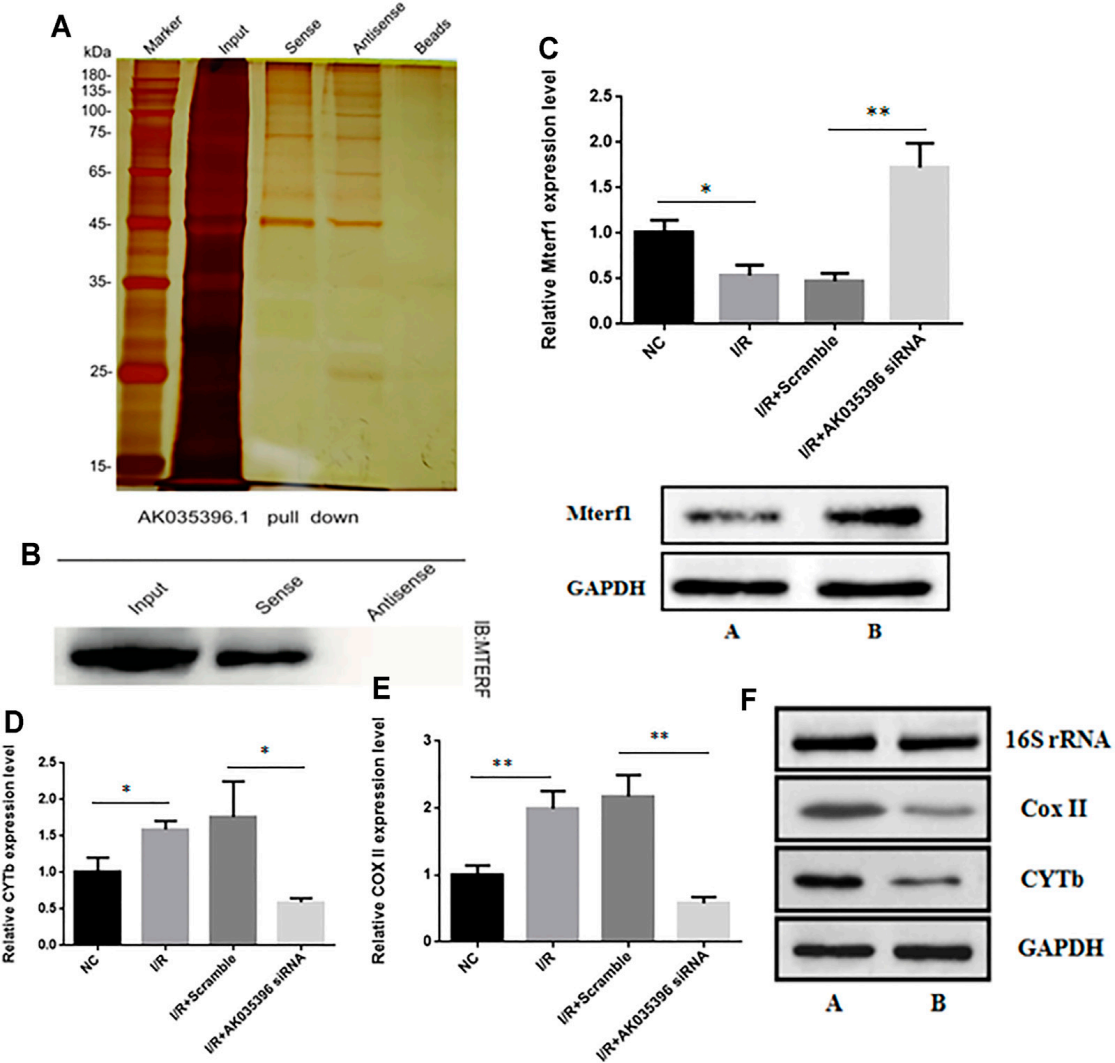
The effect of AK035396 on the expression of mitochondrial genes and target genes. Mice and primary cardiomyocytes were used to establish I/R models. **(A–B)** RNA pull-down and Western blot analyses showing that AK035396 binds to Mterf1. **(C)** The expression level of Mterf1 in the NC, I/R, I/R + Scramble and I/R + AK035396 siRNA groups were determined by qRT-PCR**(up)** and the expression of Mterf1 in the I/R group [AK035396+/+ **(A)** and AK035396−/−**(B)**] were determined by Western blotting **(down)**. **(D–E)** Expression levels of CoxII and CYTb in the NC, I/R, I/R + Scramble and I/R + AK035396 siRNA groups. **(F)** The expression of CoxII and CYTb in the I/R group [AK035396+/+ **(A)** and AK035396−/−**(B)**] was determined by Western blotting.

To further explore the mechanism by which AK035396 regulates apoptosis, we conducted RNA pulldown assays for AK035396 and found that AK035396 could specifically bind to the mitochondrial transcription termination factor Mterf1 (Figures 4A,B). We hypothesize that AK035396 may promote apoptosis by regulating the transcription of mitochondrial apoptosis-related genes. First, we first proved that AK035396 could regulate Mterf1. We verified the expression of Mterf1 in primary cardiomyocytes from neonatal rats by qRT–PCR and found that in the I/R group, the expression of Mterf1 was significantly downregulated, but after AK035396 was knocked down, the expression of Mterf1 increased (Figure 4C). Similarly, WB analysis showed the same results (Figure 4C). Subsequently, we verified the expression of the apoptosis-related proteins COXII and CYTb in mitochondria. The results showed that the expression of COXII and CYTb was upregulated after ischaemia-reperfusion. When AK035396 was knocked down, the expression of COXII and CYTb decreased significantly (Figures 4D,E). WB analysis showed the same results (Figure 4F). Overall, these results indicate that AK035396 inhibits the function of Mterf1 by binding to Mterf1 and further promotes the expression of mitochondrial COXII and CYTb, leading to apoptosis.

## Discussion

Ischaemia-reperfusion injury is an important factor that affects the prognosis of myocardial infarction (Hausenloy and Yellon, 2013). Apoptosis is an important characteristic of myocardial reperfusion injury. Therefore, it is necessary to find an effective molecular mechanism to improve myocardial cell apoptosis after ischaemia-reperfusion. The current study shows that lncRNAs are important molecules that regulate I/R in vascular organs (Zeng et al., 2019; Hu et al., 2020; Su et al., 2021). Knockdown of lncRNA AK139328 can reduce myocardial ischemia/reperfusion injury in diabetic mice (Yu et al., 2018). LncRNA MALAT1 regulates cerebral ischemia-reperfusion injury through miR-145 (Wang et al., 2020). Therefore, it is very important to clarify the biological function of lncRNAs in myocardial I/R in clinical practice to improve myocardial ischaemia-reperfusion injury. Previous studies did not prove that AK035396 is related to MIRI. We report that the regulatory role of lncRNA AK035396 in cardiomyocytes is significant for apoptosis after hypoxia/reperfusion injury. We found that knocking down AK035396 can inhibit the apoptosis of primary cardiomyocytes in I/R mice.

The role of lncRNA in organisms is indirect. It usually regulates another key factor before, during or after transcription, and further regulates the downstream function of this factor (Dykes and Emanueli, 2017). The sponge action of lncRNA and MicroRNA is a classic mode of action (Ballantyne et al., 2016; Huang, 2018). LncRNA competes with the target mRNA of miRNA in a base complementary pairing manner to reduce the content of free miRNA, thereby realizing the regulation of target mRNA. At the same time, after lncRNA binds to miRNA, it also as a target gene of miRNA, miRNA reduces the stability of lncRNA and promotes its degradation. Our results show that lncRNA AK035396 does not competitively bind to microRNA. It may regulate post-transcription and bind to mitochondrial transcription termination factors, causing Mterf1 to fail to terminate the transcription of mitochondrial genes.

Current studies have shown that the antioxidant capacity of cardiomyocytes is further reduced during ischaemia-reperfusion, and cardiomyocytes are more sensitive to reactive oxygen species than other cell types (Wu et al., 2018). The important respiratory chain protein cytochrome c oxidase family (COX) is the terminal enzyme in the mitochondrial oxidative respiratory chain, which has important functions in electron transfer and plays a critical role in oxidative metabolism, oxidation and phosphorylation in cells. The transcription of cytochrome coxidase may lead to low levels of ribose and nucleic acid production, resulting in impaired mitochondrial energy production and insufficient ATP production for respiration, leading to apoptosis (Kalpage et al., 2019).

In this study, myocardial ischaemia-reperfusion injury models were established *in vivo* and *in vitro*. The results showed that after siRNA-mediated downregulation of AK035396 in cardiomyocytes, the expression of Mterf1 was upregulated. The RNA pulldown results further confirmed that AK035396 and Mterf1 played a posttranscriptional regulatory roles. The effects of lncRNAs as regulatory factors have been confirmed in a large number of studies. Therefore, lncRNAs are considered to be important molecular functional frameworks. Through the discrete domains of secondary structures, lncRNAs can regulate the functions of different proteins, thereby improving the interactions between proteins. Since many lncRNAs are folded, transcriptional differences in exons can change the function of the carrier protein and target protein complex. lncRNAs can also act as molecular guides, acting on their neighbouring genes in a cis manner or through RNA-DNA, RNA-RNA and RNA-protein interactions to remotely affect gene translocation and guide modified protein complexes. In this study, AK035396 and Mterf1 were shown to belong to the RNA-protein regulation category. In cardiomyocytes with AK035396 downregulation, Mterf1 was no longer affected by AK035396 due to artificial knockout of the influencing factor AK035396, and the expression level of Mterf1 increased. Mterf1 can specifically bind to the 28 bp sequence after the 16S rRNA gene in mitochondrial DNA before the tRNALeu (UUR) gene, inhibiting heavy chain transcription and causing early termination. Therefore, the expression levels of several key oxidative respiration enzymes after the mitochondrial body chain are also reduced, including the apoptosis-related proteins COXII and CYTb.

## Conclusion

**FIGURE 5:**
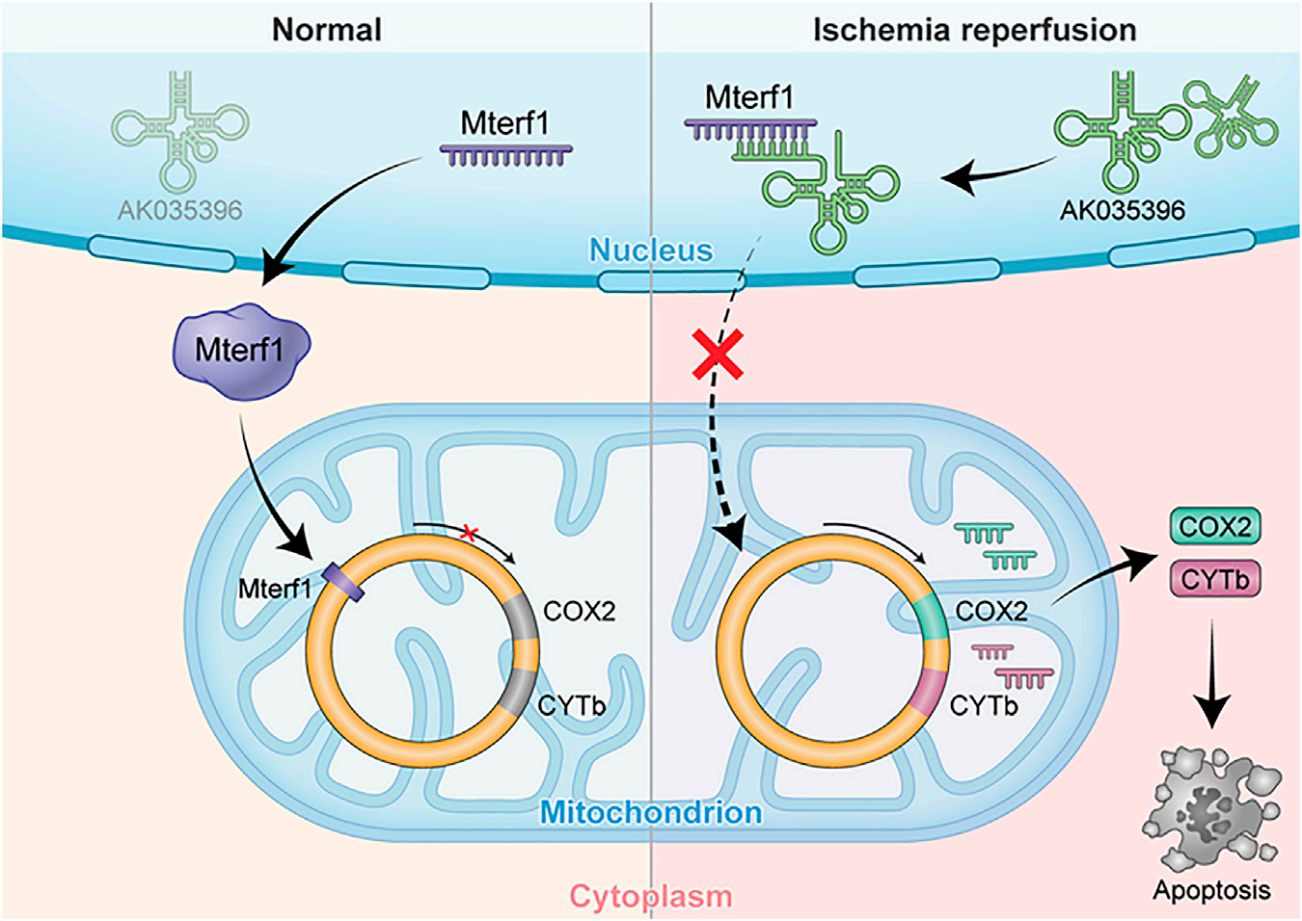
AK035396 induced myocardial apoptosis by competitively inhibiting Mterf1 and promoting the expression of mitochondrial apoptosis-related proteins COX II and CYTb after ischemia-reperfusion.

In conclusion, our research shows that the AK035396/Mterf1/COXII/CYTb axis is involved in the modulation of apoptosis in myocardial I/R (Figure 5). These findings provide the first evidence that AK035396 regulates cardiomyocyte apoptosis through mitochondrial-related pathways during myocardial I/R. Therefore, this study shows that AK035396 may be a key regulator of myocardial I/R, and inhibiting the expression of AK035396 may be a potential therapeutic strategy for myocardial I/R.

## Data Availability

Gene expression data for all samples used in this study have been uploaded in dryad (http://dx.doi.org/10.5061/dryad.rv15dv48r). URL: https://datadryad.org/stash/share/Lqs3AXs1IXN7Qo3UTXyHFQ43sKF-RVlmJ1Lxjxwczrc.

## References

[B1] Asin-Cayuela J. Gustafsson C. M. (2007). Mitochondrial Transcription and its Regulation in Mammalian Cells. Trends Biochem. Sci. 32, 111–117. 10.1016/j.tibs.2007.01.003 17291767

[B2] Ballantyne M. McDonald R. Baker A. (2016). lncRNA/MicroRNA Interactions in the Vasculature. Clin. Pharmacol. Ther. 99, 494–501. 10.1002/cpt.355 26910520 PMC4881297

[B3] Chen G. Dai J. Tan S. Meng S. Liu Z. Li M. (2014). MTERF1 Regulates the Oxidative Phosphorylation Activity and Cell Proliferation in HeLa Cells. Acta Biochim. Biophys. Sin. (Shanghai) 46, 512–521. 10.1093/abbs/gmu029 24777141

[B4] Domingos P. M. Steller H. (2007). Pathways Regulating Apoptosis during Patterning and Development. Curr. Opin. Genet. Develop. 17, 294–299. 10.1016/j.gde.2007.05.009 PMC1989756 17629474

[B5] Dykes I. M. Emanueli C. (2017). Transcriptional and Post-transcriptional Gene Regulation by Long Non-coding RNA. Genomics Proteomics Bioinformatics. 15, 177–186. 10.1016/j.gpb.2016.12.005 28529100 PMC5487525

[B6] Falkenberg M. (2018). Mitochondrial DNA Replication in Mammalian Cells: Overview of the Pathway. Essays Biochem. 62, 287–296. 10.1042/EBC20170100 29880722 PMC6056714

[B7] Fernandez-Silva P. Martinez-Azorin F. Micol V. Attardi G. (1997). The Human Mitochondrial Transcription Termination Factor (mTERF) Is a Multizipper Protein but Binds to DNA as a Monomer, with Evidence Pointing to Intramolecular Leucine Zipper Interactions. EMBO J. 16, 1066–1079. 10.1093/emboj/16.5.1066 9118945 PMC1169706

[B8] Hausenloy D. J. Yellon D. M. (2013). Myocardial Ischemia-Reperfusion Injury: a Neglected Therapeutic Target. J. Clin. Invest. 123, 92–100. 10.1172/JCI62874 23281415 PMC3533275

[B9] Hillen H. S. Temiakov D. Cramer P. (2018). Structural Basis of Mitochondrial Transcription. Nat. Struct. Mol. Biol. 25, 754–765. 10.1038/s41594-018-0122-9 30190598 PMC6583890

[B10] Hu Y.-H. Sun J. Zhang J. Hua F.-Z. Liu Q. Liang Y.-P. (2020). Long Non-coding RNA ROR Sponges miR-138 to Aggravate Hypoxia/reoxygenation-Induced Cardiomyocyte Apoptosis *via* Upregulating Mst1. Exp. Mol. Pathol. 114, 104430. 10.1016/j.yexmp.2020.104430 32240614

[B11] Huang Y. (2018). The Novel Regulatory Role of lncRNA-miRNA-mRNA axis in Cardiovascular Diseases. J. Cel. Mol. Med. 22, 5768–5775. 10.1111/jcmm.13866 PMC6237607 30188595

[B12] Kalpage H. A. Bazylianska V. Recanati M. A. Fite A. Liu J. Wan J. (2019). Tissue‐specific Regulation of Cytochrome C by post‐translational Modifications: Respiration, the Mitochondrial Membrane Potential, ROS, and Apoptosis. FASEB J. 33, 1540–1553. 10.1096/fj.201801417R 30222078 PMC6338631

[B13] Li Y. Liu X. (2018). Novel Insights into the Role of Mitochondrial Fusion and Fission in Cardiomyocyte Apoptosis Induced by Ischemia/reperfusion. J. Cel. Physiol. 233, 5589–5597. 10.1002/jcp.26522 29528108

[B14] Martin M. Cho J. Cesare A. J. Griffith J. D. Attardi G. (2005). Termination Factor-Mediated DNA Loop between Termination and Initiation Sites Drives Mitochondrial rRNA Synthesis. Cell 123, 1227–1240. 10.1016/j.cell.2005.09.040 16377564

[B15] Qian X. Zhao J. Yeung P. Y. Zhang Q. C. Kwok C. K. (2019). Revealing lncRNA Structures and Interactions by Sequencing-Based Approaches. Trends Biochem. Sci. 44, 33–52. 10.1016/j.tibs.2018.09.012 30459069

[B16] Reed G. W. Rossi J. E. Cannon C. P. (2017). Acute Myocardial Infarction. The Lancet 389, 197–210. 10.1016/S0140-6736(16)30677-8 27502078

[B17] Shi J. Ren C. Liu H. Wang L. Zhu B. Huang W. (2015). An ESRG-Interacting Protein, COXII, Is Involved in Pro-apoptosis of Human Embryonic Stem Cells. Biochem. Biophysical Res. Commun. 460, 130–135. 10.1016/j.bbrc.2015.02.130 25748575

[B18] Su Q. Lv X.-W. Xu Y.-L. Cai R.-P. Dai R.-X. Yang X.-H. (2021). Exosomal LINC00174 Derived from Vascular Endothelial Cells Attenuates Myocardial I/R Injury *via* P53-Mediated Autophagy and Apoptosis. Mol. Ther. - Nucleic Acids 23, 1304–1322. 10.1016/j.omtn.2021.02.005 33717651 PMC7920812

[B19] Terzioglu M. Ruzzenente B. Harmel J. Mourier A. Jemt E. López M. D. (2013). MTERF1 Binds mtDNA to Prevent Transcriptional Interference at the Light-Strand Promoter but Is Dispensable for rRNA Gene Transcription Regulation. Cel Metab. 17, 618–626. 10.1016/j.cmet.2013.03.006 23562081

[B20] Wang H. Zheng X. Jin J. Zheng L. Guan T. Huo Y. (2020). LncRNA MALAT1 Silencing Protects against Cerebral Ischemia-Reperfusion Injury through miR-145 to Regulate AQP4. J. Biomed. Sci. 27, 40. 10.1186/s12929-020-00635-0 32138732 PMC7059719

[B21] Wang X. Yan M. Zhao L. Wu Q. Wu C. Chang X. (2016). Low-Dose Methylmercury-Induced Apoptosis and Mitochondrial DNA Mutation in Human Embryonic Neural Progenitor Cells. Oxidative Med. Cell Longevity 2016, 1–10. 10.1155/2016/5137042 PMC4972916 27525052

[B22] Wei G. H. Wang X. (2017). lncRNA MEG3 Inhibit Proliferation and Metastasis of Gastric Cancer *via* P53 Signaling Pathway. Eur. Rev. Med. Pharmacol. Sci. 21, 3850–3856. 28975980

[B23] Wong R. S. (2011). Apoptosis in Cancer: from Pathogenesis to Treatment. J. Exp. Clin. Cancer Res. 30, 87. 10.1186/1756-9966-30-87 21943236 PMC3197541

[B24] Wu M.-Y. Yiang G.-T. Liao W.-T. Tsai A. P.-Y. Cheng Y.-L. Cheng P.-W. (2018). Current Mechanistic Concepts in Ischemia and Reperfusion Injury. Cell. Physiol. Biochem. 46, 1650–1667. 10.1159/000489241 29694958

[B25] Yu S. Y. Dong B. Fang Z. F. Hu X. Q. Tang L. Zhou S. H. (2018). Knockdown of Lnc RNA AK 139328 Alleviates Myocardial Ischaemia/reperfusion Injury in Diabetic Mice *via* Modulating miR‐204‐3p and Inhibiting Autophagy. J. Cel. Mol. Med. 22, 4886–4898. 10.1111/jcmm.13754 PMC6156366 30047214

[B26] Zeng J. Zhu L. Liu J. Zhu T. Xie Z. Sun X. (2019). Metformin Protects against Oxidative Stress Injury Induced by Ischemia/Reperfusion *via* Regulation of the lncRNA-H19/miR-148a-3p/Rock2 Axis. Oxidative Med. Cell Longevity 2019, 1–18. 10.1155/2019/8768327 PMC6942897 31934270

[B27] Zhao W. Geng D. Li S. Chen Z. Sun M. (2018). LncRNA HOTAIR Influences Cell Growth, Migration, Invasion, and Apoptosis *via* the miR-20a-5p/HMGA2axis in Breast Cancer. Cancer Med. 7, 842–855. 10.1002/cam4.1353 29473328 PMC5852357

[B28] Zidan A. Awaisu A. Kheir N. Mahfoud Z. Kaddoura R. AlYafei S. (2016). Impact of a Pharmacist-Delivered Discharge and Follow-Up Intervention for Patients with Acute Coronary Syndromes in Qatar: a Study Protocol for a Randomised Controlled Trial. BMJ Open 6, e012141. 10.1136/bmjopen-2016-012141 PMC5129077 27864247

